# Calculated parenteral initial treatment of bacterial infections: Infections of the kidneys and the genito-urinary tract

**DOI:** 10.3205/id000056

**Published:** 2020-03-26

**Authors:** Reinhard Fünfstück, Udo Hoyme, Kurt Naber, Adrian Pilatz, Sören Schubert, Florian Wagenlehner

**Affiliations:** 1 Klinik für Innere Medizin, Sophien- und Hufeland-Klinikum gGmbH Weimar, Germany; 2 Klinik für Gynäkologie und Geburtshilfe St. Georg Klinikum Eisenach, Germany; 3 Urologische Klinik und Poliklinik, Technische Universität München, Munich, Germany; 4 Klinik für Urologie, Kinderurologie und Andrologie, Justus Liebig Universität Giessen, Germany; 5 Max von Pettenkofer-Institut, Medizinische Fakultät, Ludwig Maximilians-Universität München, Munich, Germany

## Abstract

This is the eighth chapter of the guideline “Calculated initial parenteral treatment of bacterial infections in adults – update 2018” in the 2^nd^ updated version. The German guideline by the Paul-Ehrlich-Gesellschaft für Chemotherapie e.V. (PEG) has been translated to address an international audience.

The chapter deals with the treatment of more severe infections of the kidney and the urogenital tract, including urosepsis. Recommendations for empiric and targeted antibacterial treatment are given.

## Zusammenfassung

Dies ist das achte Kapitel der von der Paul-Ehrlich-Gesellschaft für Chemotherapie e.V. (PEG) herausgegebenen S2k Leitlinie „Kalkulierte parenterale Initialtherapie bakterieller Erkrankungen bei Erwachsenen – Update 2018“ in der 2. aktualisierten Fassung.

Es befasst sich mit der Behandlung schwererer Infektionen der Niere und des Urogenitaltrakts einschließlich der Urosepsis und enthält Empfehlungen für eine empirische und gezielte antibakterielle Behandlung.

## Indication for initial parenteral antibiotic treatment

For kidney and genito-urinary tract infections, initial (empirical) parenteral antibiotic treatment is usually only required in severe clinical cases with general symptoms, such as nausea and vomiting or suspected sepsis [1]. These are mainly severe forms of uncomplicated or complicated or nosocomial pyelonephritis, acute prostatitis, rarely acute epididymitis with or without orchitis, acute salpingitis-pelvioperitonitis or severe abscessing infections in the kidney and genito-urinary tracts. Occasionally, parenteral treatment must also be initiated empirically if multidrug-resistant agents for which no oral antibiotics are available must be expected and waiting for microbiological test results is not an option, for example because surgery must be performed immediately (e.g. in acute urinary stone obstruction).

## General criteria for antibiotic selection

The choice of antibiotics (Table 1 (Tab. 1)) is made in accordance with the expected pathogen spectrum, taking into account pharmacokinetic and pharmacodynamic aspects. In any case, ensuring sufficient renal elimination of the drug should be considered for the treatment of infections of the urinary tract [2], [3]. In addition, the so-called collateral damage of antibiotics should be considered, which goes beyond the side effects in the individual patient and favors resistance development or the selection of resistant pathogens (see chapter 2 [4], paragraph collateral damage of antibiotics). In addition to antibiotic treatment, appropriate general as well as subject-specific therapeutic measures are to be taken but these are not the subject of this article [5], [6], [7].

## Acute uncomplicated pyelonephritis

The most common pathogen is *Escherichia* *coli*, followed by *Proteus* *mirabilis* and *Klebsiella* *pneumoniae* [8], [9], [10]. Rarely, other Enterobacteriaceae are detected in the urine. Larger epidemiological studies on pathogen sensitivity are missing. However, studies on uncomplicated cystitis can be used because it has approximately the same pathogen spectrum although *Staphylococcus* *saprophyticus* occurs less frequently and the resistance situation is roughly the same [11]. Renal parenchymal damage may possibly be prevented by initiating effective treatment in time. Initial (empirical) parenteral treatment with a group 3a cephalosporin, an aminopenicillin/beta-lactamase inhibitor (BLI), an aminoglycoside (not a first choice due to the risk of side effects, always in combination with other antibiotics) is indicated whenever severe general symptoms with nausea and vomiting are present [1], [12], [13], [14], [15], [16], [17]. Fluoroquinolones with high renal excretion, such as ciprofloxacin or levofloxacin may be given if fluoroquinolone resistance is unlikely. After the symptoms improve, oral treatment should replace parenteral treatment in line with the test results as soon as possible. This should be a suitable oral fluoroquinolone, for example ciprofloxacin or levofloxacin, an oral cephalosporin of group 3 (cefpodoxime), an aminopenicillin in combination with a BLI or cotrimoxazole or trimethoprim; but only if the pathogen has been tested as sensitive [1], [18], [19], [20]. The duration of treatment is based on the clinical progression, usually 7 days are sufficient [21]. 

## Complicated or nosocomial urinary tract infection

### Definition

A complicated urinary tract infection (UTI) is defined as an infection of the urinary tract that is associated with a morphological, functional or metabolic anomaly, which leads to disturbance of renal function, impaired urinary transport and disorder of local and systemic defense mechanisms [5], [22], [23], [24]. The risk factors are classified in the so-called ORENUC classification [25].

### Indication for initial parenteral antibiotic treatment

The indication for initial parenteral antibiotic treatment, as mentioned above, depends on the general condition and the risk profile of the patient. CRP and procalcitonin can be used as biomarkers in the decision making process [26]. Antibiotic treatment of complicated UTIs can only achieve a cure if the complicating or triggering factors are eliminated or improved [1].

### Pathogen spectrum

The expected pathogen spectrum is generally much broader than that of uncomplicated UTIs and is also related to the circumstances in which a complicated UTI was acquired [22], [23], [27]. So, the pathogen spectrum in community-acquired first-time complicated UTIs, for instance as a result of acute calcium oxalate urinary stones in a patient with no prior antibiotic and no urinary diversion, is relatively similar to the spectrum of an uncomplicated acute pyelonephritis [6]. But in cases of nosocomially acquired complicated UTIs, pathogens which are not usually pathogens of primary urinary tract infections but appear secondarily as a result of selection or colonization should also be expected. These may be *Pseudomonas* *aeruginosa* or Enterobacteriaceae like *Escherichia* *coli* [23], [27]. If a complicated UTI is suspected, a urine culture prior to the initiation of antibiotic treatment is generally indicated because the pathogen spectrum is broad and the resistance situation not always predictable, so options for adapting treatment following the microbiological test result should be considered [1].

### Antibiotic selection

The initial empirical antibiotic treatment must take into account the regional resistance situation of the expected pathogen spectrum [23]. The following clinical conditions which have an impact on the expected pathogen spectrum and pathogen sensitivity must always be clarified in advance [23]:

1. Where was the UTI acquired, for instance as an out-patient, in a nursing home, hospital, after diagnostic/therapeutic interventions?
2. Was there prior antibiotic treatment (for how long, what antibiotics)?
3. Was there a previous longer hospital stay?
4. Was there a previous urinary diversion (what, how long, treated how)?
5. If urinary diversion is present, check the quality of the urinary drainage and, if necessary, change the catheter (removal of the infectious biofilm)
6. Is it a case of recurrence or treatment failure?
7. For antimicrobial stewardship reasons, consideration should always be given to the extent to which the use of broad spectrum antibiotics is necessary (such as cephalosporins/BLI, carbapenems).

For initial parenteral treatment of complicated complex UTI, cephalosporins of group 3a, fluoroquinolones and aminopenicillins/BLI are suitable. In the case of risk factors for multidrug-resistant pathogens (such as extended spectrum beta-lactamase [ESBL]-producing Enterobacteriaceae), antibiotics such as cephalosporins/BLI (ceftolozane/tazobactam, ceftazidime/avibactam) or a carbapenem of group 2 (ertapenem) may be used [1], [8], [9], [10], [12], [13], [14], [15]. Patients with nosocomially acquired or catheter-associated UTIs also show an increased incidence of multidrug-resistant pathogens [23], [24], [27], [28]. An antibiotic which is also effective against more rare and multi-resistant Gram-negative pathogens should therefore be used for empirical treatment. These include cephalosporins of group 3b, the cephalosporin/BLI combinations ceftolozane/tazobactam and ceftazidime/avibactam, or 4 (cefepime), fluoroquinolones of group 2 or 3 (observe local *Escherichia* *coli* resistances) and carbapenems of group 1 (imipenem, meropenem) [1], [9], [29], [30]. To close the enterococcal gap present in these antibiotics as mixed infections with enterococci are more common in catheter-associated urinary tract infections, acylaminopenicillin/BLI (such as piperacillin/Tazobatam) should be used [1], [31]. If multidrug-resistant pathogens are suspected (in the context of outbreaks or high endemic resistance rates), appropriately effective substances should be used in empirical treatment. Since carbapenemases rarely occur in German-speaking countries, a cephalosporin/BLI (ceftolozane/tazobactam, ceftazidime/avibactam) [8], [9], [10], a carbapenem of group 2 (ertapenem) or, in case of simultaneous suspected Pseudomonas, a cephalosporin/BLI (ceftolozane/tazobactam, ceftazidime/avibactam) or a carbapenem of group 1 (imipenem, meropenem) [1], [8], [9], [10], [29], [30] are suitable. In order to conserve carbapenems and thus counteract the selection of carbapenem-resistant pathogens, the new cephalosporin/BLI combinations (ceftolozane/tazobactam, ceftazidime/avibactam) are also suitable for parenteral initial treatment [8], [9]. For ESBL-producing pathogens, fosfomycin might also be considered for parenteral initial treatment; however, there is limited data on fosfomycin monotherapy in complicated UTIs [32], [33]. For the treatment of infections with methicillin-resistant *Staphylococcus* *aureus* (MRSA) and vancomycin-resistant enterococci (VRE) several highly-effective substances are now available, such as ceftobiprole (effective only against *Staphylococcus* *aureus* and some strains of *Enterococcus* *faecalis)*, daptomycin (effective only against enterococci in high, unauthorized doses) or linezolid [2], [34], [35]. For urinary tract infections, however, there are insufficient studies, so that case-by-case decisions on treatment are necessary. 

### Patients with diabetes mellitus

Urinary tract infections in patients with diabetes mellitus are problematic because they can increase the pathogenetically significant insulin resistance and worsen an unstable metabolic situation by activating inflammatory processes. This is especially true for patients with a HbA1c value >8.5% (HbA1c – IDFF >70 mmol/l) with a tendency to hypo- and hyperglycaemia, for patients with a BMI >30 kg/m^2^ and for cases with a manifest diabetic nephropathy (from stage 2b, albumin excretion ≥200 mg/l, creatinine clearance ≤60 ml/min) [36]. Glucosuria promotes colonization of the urinary tract by pathogenic and facultative pathogenic microorganisms. 

In case of asymptomatic bacteriuria, no antimicrobial treatment is necessary in a stable diabetic metabolic situation and if obstructive disorders and other anatomical changes have been excluded [37], [38]. For both uncomplicated and complicated infections, the same treatment recommendations are applicable for patients with or withour diabetes mellitus. This is true for the initial parenteral as well as the oral follolw-up treatment. It should be noted that antimicrobial chemotherapeutic agents may increase the hypoglycemic effect of oral anti-diabetic drugs; however other interactions between antibiotics and anti-diabetic drugs are rare.

### Patients with impaired renal function and after kidney transplantation

Urinary tract infections favor the progression of acute and chronic renal failure. In patients with renal impairment, dialysis or renal transplantation, no potentially nephrotoxic antibiotics should be used, such as aminoglycosides or vancomycin. The dosage of antibiotics depends on the degree of renal impairment. Dosage recommendations can be found in nomograms or tables with detailed breakdowns [39], [40], [41] as pharmacokinetic parameters of the antimicrobial substances depend extensively on different excretion modes of each substance and the respective renal function (see corresponding tables) [39], [40], [41], [42], [43], [44], [45], [46], [47].

Under the conditions of renal replacement therapy, pharmacological properties such as the molecular size of the antibiotic, its solubility in water and protein binding and the dialysis procedure influence dosage recommendations. It makes sense to only administer antibiotics after completion of the dialysis (HD, HDF, CVVHD, CVVHDF).

## Urosepsis

Urosepsis occurs after hematogenous spread from the infected urinary tract without or following instrumental intervention. Mainly *Escherichia* *coli* and other Enterobacteriaceae are found. After urological intervention or in patients with urinary diversion via a catheter, multidrug-resistant *Pseudomonas* spp., *Proteus* spp., *Serratia* spp., *Enterobacter* spp., enterococci and staphylococci must also be considered (see complicated UTI) [1], [23]. 

Initial parenteral antibiotic treatment must be initiated immediately (within the first hour) in cases of suspected urosepsis and after prior sampling of urine and blood cultures [48], [49], [50], [51], [52] , group 3 or 4 cephalosporins are the most useful for this [13], [53], [54]. Alternatives are acylaminopenicillin/BLI (such as piperacillin/tazobactam) or cephalosporin/BLI (ceftolozan/tazobactam, ceftazidime/avibactam) or a carbapenem of group 2 (ertapenem) or group 1 (imipenem, meropenem), depending on the circumstances under which urosepsis has occurred (see complicated/nosocomial UTI) [8], [9], [10], [13], [29], [30], [31]. An extension of the antibacterial spectrum can initially be achieved, for example through combination with an aminoglycoside or a fluoroquinolone with high urinary elimination [1]. In general, the highest possible dosage of antibiotics should be chosen [3], [55].

Urosepsis usually involves an obstructive uropathy (for example due to urolithiasis, tumors, benign prostatic hypertrophy or where an abscess-forming infection is present). Therefore, after taking samples (urine, blood) and initiating an appropriate broad-spectrum antibiotic treatment, in addition to necessary intensive care measures in case of sepsis, targeted urological diagnostics must be performed immediately to detect or localize the obstructive uropathy or the abscessing infection. The aim is to eliminate the source as quickly as possible or to circumvent the obstruction with suitable measures (such as a transurethral or suprapubic catheter, ureteral splint or nephrostomy) to allow free urine drainage once more [48], [49], [56], [57].

## Acute prostatitis, prostate abscess

Empirical treatment of acute bacterial prostatitis (ABP) is based on the same aspects as those for complicated urinary tract infections [1], [58]. In spontaneously occurring ABP, mainly *Escherichia* *coli* and other Enterobacteriaceae are found. In patients with ABP following urological intervention, other Gram-negative pathogens, for example *Pseudomonas* spp. are commonly found. In these patients, ABP is more often associated with abscess-forming progression; here *Klebsiella* *pneumoniae* are again found more frequently [59].

For empirical treatment, the preference is for substances which, in addition to high antibiotic concentrations in the urine, also ensure sufficient concentration in the prostate tissue, prostatic fluid and ejaculate [1]. Initial parenteral antibiotic treatment is only necessary in severe forms of acute bacterial prostatitis with or without abscess formation. The drugs of choice are fluoroquinolones of groups 2 and 3 [58], [59], [60]. However, an antibiotic history is especially important as many patients have previously received fluoroquinolones and the risk of fluoroquinolone-resistant pathogens is high. Alternatively, it is also possible to use groups 3 and 4 cephalosporins or acylaminopenicillins/BLI in an ABP. Since ABP is not a common infection but antibiotic treatment has to be initiated immediately, there are no prospective, controlled or randomized studies, so the treatment recommendations are essentially based on expert opinions [1], [58], [61].

Following in-culture pathogen identification from the urine (prostate massage is contraindicated in ABP) and the results of resistance testing, treatment should be switched to targeted therapy, depending on the improvement of the clinical situation and continued orally for at least two (up to four) weeks, to avoid complications such as acute urinary retention, epididymitis, prostate abscess or chronic prostatitis [1], [58], [61].

## Acute epididymitis, epididymoorchitis possibly with abscess

While an etiological age limit of 35 years has traditionally been set (<35 years: sexually transmitted pathogens, >35 years for classical uropathogens) [1], [62], it has recently been shown that classical uropathogens are also common in young patients, and the antimicrobial makeup must therefore be adequately assessed [63], [64].

This means that, with the exception of gonococcal epididymitis, in young men preferably fluoroquinolones with activity against chlamydia (for example levofloxacin), should be used. The combination of ceftriaxone i.m. with doxycycline p.o. is still accepted for sexually transmitted pathogens. In older men, group 2 and 3 fluoroquinolones are also options if out-patient treatment is possible [64]. 

Generally, parenteral and in-patient treatment is necessary only for severe forms (for example abscess formation), severe comorbidities (for example indwelling catheters) or treatment failures. Note there is an increasing resistance to fluoroquinolones, especially amongst catheter carriers, so that combination therapy makes sense, for example with a group 3a cephalosporin. Once the antibiogram has been created, parenteral treatment should be switched to oral sequential therapy as soon as possible [63].

## Endometritis, salpingitis, tubo-ovarian abscess, pelvic peritonitis

A broad spectrum of potential pathogens must be expected for infections of the genitalia of sexually active pre-menopausal women. In addition to sexually transmitted pathogens *Neisseria* *gonorrhoeae* and *Chlamydia* *trachomatis*, etiologically the vaginal flora and pathogens of bacterial vaginosis must be considered, in rare cases also mycoplasmas and ureaplasmas [65], [66], [67]. With regard to the etiology of ascended infections, with exceptions only laparoscopic samples are diagnostically relevant [68]. Since no single antibiotic is effective against the full spectrum of possible pathogens, no consensus on the treatment of choice has yet been found, especially as a variety of studies with combinations of different substance groups have shown positive results. Unequivocal evaluation of parenteral and oral treatment regimes as well as comparisons of out-patient and in-patient treatment has not been carried out to date, so choosing a treatment must be made on a case-by-case basis according to the severity of the disease, patient acceptance and regional resistance of potential pathogens. After clinical improvement, initial parenteral therapy can be switched to oral sequential therapy with one of the combination partners, usually doxycycline, clindamycin or even a fluoroquinolone [62], [69]. Cephalosporins should be combined primarily with metronidazole to cover anaerobes. Alternatively, fluoroquinolones or aminopenicillins/BLI can be used. Fluoroquinolones of groups 2 and 3 are used, in each case in combination with metronidazole, aminopenicillins/BLI plus doxycycline or a carbapenem of group 2 (ertapenem) [69]. Progress monitoring is always required within 72 hours, even in cases of seemingly uncomplicated infections [62]. In the event of treatment failure, antimicrobial treatment should be adapted quickly in line with the microbiological findings available and, if necessary, surgical intervention should be initiated [65], [66]. 

In pregnancy, embryotoxic or teratogenic potential of various antibiotic groups should be taken into account if used before the 14^th^ week.

The screening established in Germany for asymptomatic bacteriuria in pregnancy is under discussion once more. A Cochrane analysis based on 14 studies showed a reduction from 21% to 5% for pyelonephritis (RR 0.23, 95% CI: 0.13–0.41), birth weight <2,500 g from 13% to 8.5% (RR 0.66, 95% CI: 0.49–0.89) and not significant premature birth <38 weeks of age from 21% to 13% (RR 0.37, 95% CI: 0.10–1.36) with antibiotic treatment [70]. In contrast, a recently published prospective randomized multi-center study found no difference in the prevalence of lower birth weight and earlier time of birth but did find a difference in relation to pyelonephritis [71]. At 16–22 weeks, however, screening and possibly nitrofurantoin treatment start rather late. A more detailed analysis of newborns did not take place. In principle, as far as obstetrics are concerned, the assumption is that studies looking beyond urinary tract infections are difficult to assess and ethically very problematic or hardly feasible.

## Note

This is the eighth chapter of the guideline “Calculated initial parenteral treatment of bacterial infections in adults – update 2018” in the 2^nd^ updated version. The German guideline by the Paul-Ehrlich-Gesellschaft für Chemotherapie e.V. (PEG) has been translated to address an international audience.

## Competing interests

The authors declare that they have no competing interests.

## Figures and Tables

**Table 1:**
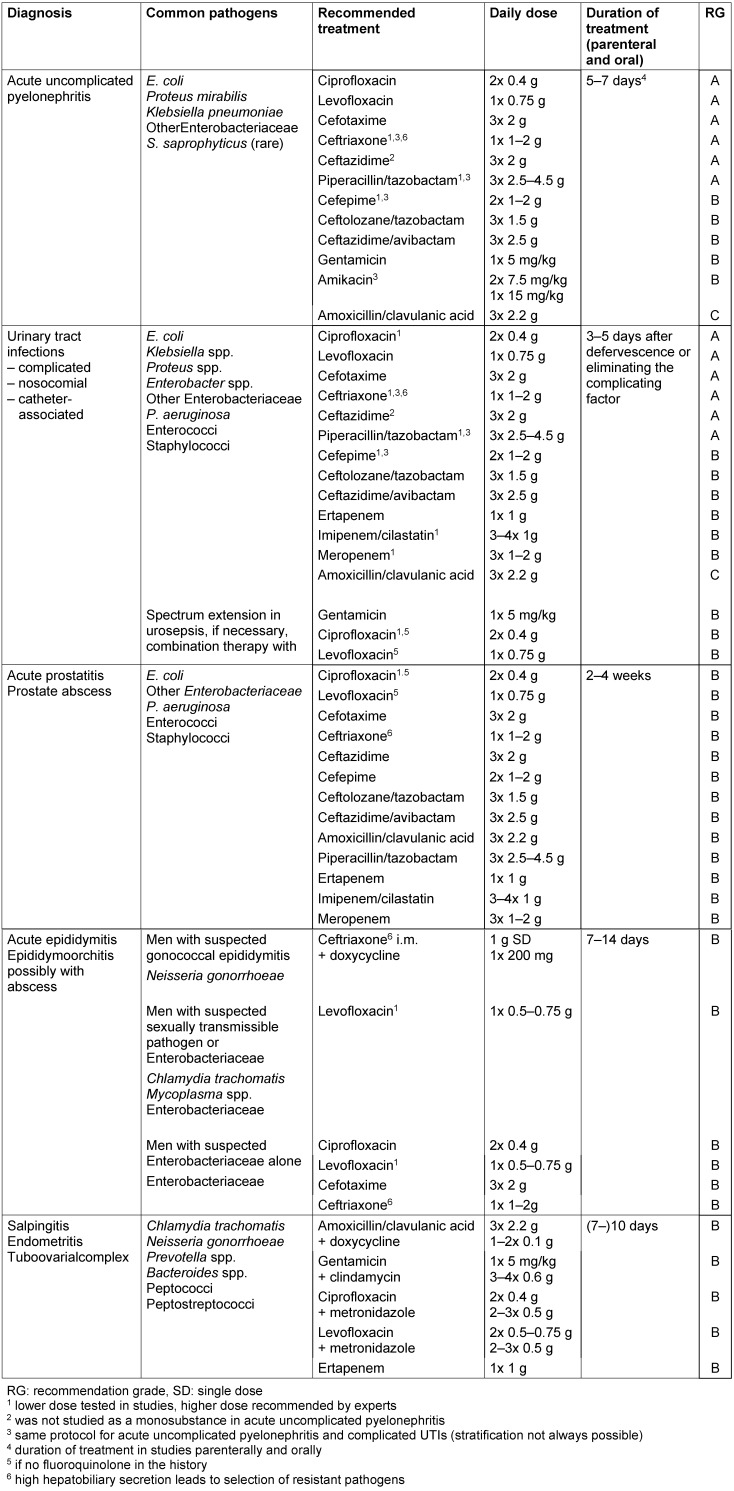
Recommendations for empirical initial parenteral antibiotic treatment for kidney and genito-urinary tract infections (antibiotics by groups)
